# Preparation and Characterization of Triglycine-Containing 3D-Printed PBAT/PLA Specimens

**DOI:** 10.1021/acsomega.5c03205

**Published:** 2025-05-26

**Authors:** Khadar Duale, Alexander Grundmann, Simon T. Kaysser, Sönke Detjen, Paweł Chaber, Jakub Włodarczyk, Henryk Janeczek, Marta Musioł, Iza Radecka, Marek Kowalczuk, Anna Hercog, Sunita Ranote, Joanna Rydz

**Affiliations:** ‡ Centre of Polymer and Carbon Materials, Polish Academy of Sciences, M. Curie-Skłodowskiej 34, Zabrze 41-800, Poland; § CompriseTec GmbH, Rödingsmarkt 20, Hamburg 20459, Germany; Φ School of Life Science, Faculty of Science and Engineering, 105674 University of Wolverhampton, Wulfruna St., Wolverhampton WV1 1LY, U.K.

## Abstract

This study explores a novel additive—0.2 wt % monodisperzed H-Gly-Gly-Gly-OH (triglycine)—to poly­(1,4-butylene adipate-*co*-1,4-butylene terephthalate)/polylactide (PBAT/PLA) blend. The triglycine-containing PBAT/PLA was extruded into uniform filaments with a diameter of 1.74 ± 0.02 mm by using a single-screw extruder, which was subsequently employed to fabricate specimens via material extrusion (MEX) technology at nozzle temperatures (*T*) of 155 and 190 °C. In the present study, the fabrication of triglycine-containing specimens was investigated with a focus on elucidating their thermal behavior and mechanical properties. The influence of print temperature on these properties was also evaluated to establish correlations between processing conditions and material performance. Results indicated that all specimens could be printed smoothly at 155 and 190 °C, regardless of triglycine presence. The addition of triglycine did not significantly affect the melting temperature of the specimens, but it did reduce the difference in glass transition temperature between PLA and PBAT. SEM analysis did not display any significant differences between the specimens across all nozzle temperatures. Specimens without triglycine displayed consistent mechanical properties across temperatures, achieving a high tensile modulus (*E*) of 261.5 MPa for specimens printed at *T* = 190 °C. Conversely, triglycine-containing specimens printed at the same temperature exhibited a significantly lower tensile modulus (*E* = 159.5 MPa). However, the triglycine inclusion enhanced the ultimate tensile strength at lower print temperatures, with values of 15.03 ± 0.11 MPa for specimens printed at *T* = 155 °C compared to 10.16 ± 1.65 MPa for specimens printed at *T* = 190 °C.

## Graphical abstract

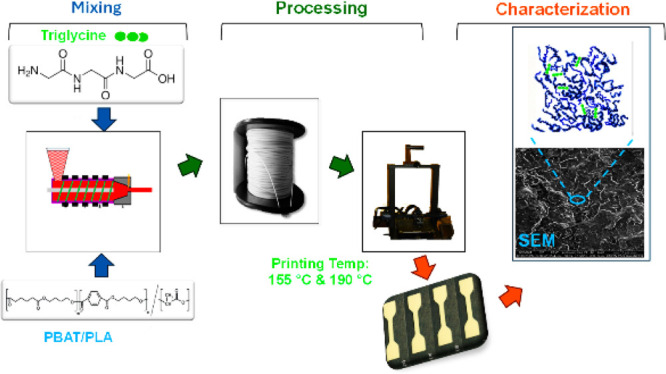

## Introduction

The rapid advances in additive manufacturing (AM), commonly referred to as three-dimensional (3D) printing, are attested by a plethora of scientific and technical publications.^1–3^ Through its innovative approach, the technology has evolved significantly since its inception, revolutionizing both the prototyping and the manufacturing industries. The 3D printing technology is now so mature that it is recognized for its ability to produce complex structures with a remarkable range of applications, including various high-value areas such as electronics, soft robots, biomedical devices, tissue engineering, food processing, and packaging.^4–8^ Among all the AM technologies, material extrusion (MEX), also known as fused deposition modeling (FDM) and fused filament fabrication (FFF), is a rapidly growing AM technology that is widely used for the fabrication of plastic materials.^9,10^ Its versatility and scalability have made it a first choice in various industries, from prototyping to production-grade manufacturing, heralding a new era of fast and cost-effective production processes.^11,12^ In short, the MEX method is based on an additive principle where a material is layered on a build platform using a plastic filament from a spool that is wound up to fabricate complex structures with remarkable precision and efficiency.^10^ The technology relies on the precise extrusion of thermoplastic filaments since melting the filament is one of the most important parts and is a common 3D printing method for thermoplastic polymers. There are numerous types of filaments commercially available on the market today, depending on the part to be manufactured and its functionality.^13^ Polymers used in AM processes are typically thermoplastic filaments, resins, or powders, and the most commonly used for MEX are polylactide (PLA) and acrylonitrile-butadiene-styrene terpolymer (ABS).^14^ Biodegradable polymers have received tremendous attention due to environmental concerns and a desire to move away from finite petroleum resources.^15^ The application of biodegradable polymers integrated into the manufacturing capabilities of 3D technology has recently started to emerge in various biomedical fields, such as tissue engineering, bone repair, and dentistry.^16–18^ This increases the demand for biodegradable alternatives in 3D printing.

Ecovio (PBAT/PLA) is a biobased blend of aliphatic–aromatic copolyester with PLA that has found a wide range of applications.^19–22^ PBAT is blended with PLA to improve its properties, such as processability, ductility, toughness, and melt strength, broadening its applications while maintaining its environmentally friendly and biodegradable nature.^23,24^ PLA/PBAT blends are highly versatile materials that have found sustainable applications in biodegradable packaging, agriculture, and biomedicine. In the packaging industry, they are used for films and bags.^25^ In agriculture, PBAT/PLA composites are used in mulch films that degrade naturally, minimizing environmental impact.^26^ PBAT/PLA containing active substances for agricultural usage have also been reported.^21,27^ Their strength and biocompatibility also make them promising for biomedical applications, especially in tissue engineering, where they fulfill the mechanical requirements of bone scaffolds while having low density and high toughness. A composite of PLA, PBAT, nanohydroxyapatite, and microcrystalline cellulose was reported for use in bone tissue.^28^ The study found that the addition of PBAT improved the interfacial compatibility and mechanical properties, without impairing the inherent biodegradability of the materials. Kang et al.^29^ developed porous PLA/PBAT scaffolds using a nonsolvent-induced phase separation method and showed that PBAT significantly improves tensile strength and creates microporous structures that enhance water uptake, coupled with pore size and porosity. These scaffolds provide an optimal environment for cell adhesion and cell proliferation assays, confirming no negative effects on cell activity and highlighting their potential in biomedical applications.

Nevertheless, despite outstanding physical and mechanical properties, research on PBAT/PLA in 3D-printed materials continues to be limited. Among the few studies done on PBAT/PLA, the effect of several parameters such as layer thickness, deposition rate, and direction of pressure on the porosity and flexural properties of PBAT/PLA parts obtained using MEX was investigated.^30^ Although a wide range of filaments, composites, and inks have been developed in recent years, the field of AM still faces challenges that limit its wider use in advanced biomedical applications. Some of the challenges are due to the scarcity of materials, while others involve transforming these materials into objects with controllable mechanics, degradation rates, and bioactivity. Besides material selection, many parameters play a crucial role in 3D printing. These are important to improve the mechanical properties of the materials. The effect of properties such as print temperature, layer thickness, printing speed, and bed temperature of MEX of PBAT/PLA with a PLA-*g*-GMA (glycidyl methacrylate) compatibilizer was also investigated.^31^ The authors concluded that the optimal mechanical properties were obtained with a layer thickness (0.15 mm), printing speed (50 mm·s^–1^), nozzle temperature (200 °C), and bed temperature of 50 °C. The miscibility of PBAT and PLA is an issue, and several papers reported different ways to improve the ductility of the blend before printing. Lyu et al.^32^ investigated the mechanical properties of MEX of PBAT/PLA specimens, where PLA was grafted with GMA. The authors concluded that the filament orientation with microchannels is assumed to be the main influencing factor. When the two printing directions were compared, the specimens printed in transverse deposition showed that PLA-*g*-GMA promoted adhesion between the filament layers. However, for AM specimens in the longitudinal direction, the mechanical properties showed much higher values than for injection-molded specimens. In a similar study, poly­(methyl methacrylate) (PMMA) was used to improve the compatibility between PLA and PBAT, and the tensile results of the AM specimens showed improved mechanical properties with the introduction of PMMA in the PBAT/PLA blends.^33^ MEX is affected by many parameters, and adjusting these parameters is an effective method to enhance interlayer adhesion and mechanical properties. However, these parameters can be altered if a substance is added to the polymer. Printing polymer impregnated with filler or a small amount of additive can completely change the printing parameters to achieve the desired properties of the polymer in its original form. More fundamental research on biodegradable PBAT/PLA blends is still necessary to address the knowledge gap on their behavior in 3D printing. This study aimed to investigate the effect of the small peptide of triglycine on the properties of sustainable PBAT/PLA blends during AM via MEX, focusing on how varying temperature conditions influence these characteristics. Triglycine is a tripeptide composed of three glycine residues linked by peptide bonds in a linear sequence. It is one of the best studied model peptides and serves as a model compound for peptide–polymer interactions and physicochemical parameter studies of small peptides. The small, detectable structure makes it ideal for tracking within polymers, while triglycine sulfate finds advanced applications in pyroelectric detection for radiation sensors and infrared spectroscopy.^34^ The current study aims to integrate triglycine into PBAT/PLA blends via an AM process and evaluate its impact on the structural and functional properties of the blend. The hypothesis is that the incorporation of triglycine into the PBAT/PLA blend will serve as an exemplary model for potential biomedical applications. The specimens could also serve as a molecular labeling agent to store binary information in a bulk mixture of polymer and oligopeptide, akin to what was previously reported for poly­(l-lactide) (PLLA).^35^ In this case, the presence of a glycine-rich oligopeptide with the [NH_2_]­GGGGGGAAGAG­[COOH] sequence was used as a defined label for marking (bio)­degradable polymer films for potential biomedical applications. For biomedical applications, it is important to evaluate the interaction between the oligopeptide additive and the polymer. For instance, the small amount of oligopeptide additive impregnated in the polymer carrier can influence the physicochemical and mechanical properties of the polymer, and that needs to be evaluated. On the other hand, some oligopeptide additives can be unstable under certain processing conditions and can degrade during material processing, such as in melt extrusion, thermosetting, and 3D printing.^10,36^ This work highlights the versatility of 3D printing as a technique to design materials with new functionalities using biodegradable polymers. In particular, PBAT/PLA, a blend of biodegradable aliphatic–aromatic copolyester with PLA, is promising for various applications, but its application in 3D printing is underexplored. This research, in addition to making the filament, investigates the incorporation of triglycine into PBAT/PLA blends. The fabrication of a PBAT/PLA 1BA-shaped standard specimen (with dimensions according to ISO 527 standard) that contains 0.2 wt % monodisperse H-Gly-Gly-Gly-OH (triglycine) was performed. The influence of triglycine and nozzle temperature on the morphology and mechanical and thermal properties of specimens is examined, comparing the results with and without the oligopeptide additive. The printed specimens were subsequently characterized by using Fourier-transform infrared spectroscopy (FTIR), scanning electron microscopy (SEM), differential scanning calorimetry (DSC), and thermogravimetric analysis (TGA).

## Experimental Section

### Materials

PBAT/PLA pellets (Ecovio F Mulch C2311), with a density of 1.22 g cm^–3^ and melt mass-flow rate (MFR) of 23.1 g·10 min^–1^ (230 °C, 2.16 kg), were used to prepare the filament. The Mulch C2311 was a commercial blend (purchased from BASF SE, Ludwigshafen, Germany) of PBAT (containing 47 mol % aromatic segments) with 12 mol % PLA, as previously reported.^22^ H-Gly-Gly-Gly-OH, (triglycine) from Sigma-Aldrich, Taufkirchen, Germany, with purity ≥98% and relative molar mass *M*_*r*_ = 189.17 g·mol^–1^ was used as received.

### Thermal Properties

The thermal properties of the initial PBAT/PLA pellets, filaments with and without triglycine, and 1BA-shaped standard specimens were analyzed through DSC using a TA Instruments Q2000 apparatus (Newcastle, DE, USA). The instrument was calibrated with a high-purity indium. The studies were carried out at a temperature from −90 to 200 °C with a rate of 20 °C·min^–1^ to eliminate the effect of thermal history (first heating run). The second heating run from −80 to 200 °C (at a heating rate of 20 °C·min^–1^) was done for samples after rapid cooling from the melt. All experiments were performed under a nitrogen atmosphere with a nitrogen flow rate of 50 mL·min^–1^, using aluminum standard sample pans. The melting temperature (*T*_*m*_) was taken as the peak temperature maximum of that melting endotherm, and the glass transition temperature (*T*_*g*_) was taken from the second heating run for the amorphous samples obtained by rapid cooling from the melt and as the midpoint of the heat capacity change of the specimen.

The characteristic thermal decomposition and stability behavior of the specimens were determined by using a TGA/SDTA 851 Mettler-Toledo thermal analyzer from room temperature to 800 °C at a heating rate of 10 °C·min^–1^ in a stream of nitrogen (60 mL·min^–1^). The obtained TGA data were analyzed using a Mettler-Toledo Star System SW 15.00. Samples of approximately 15 mg were introduced into 70 μL of aluminum oxide crucibles.

### FTIR Analysis

FTIR was recorded on a JASCO FT/IR-6700 spectrometer (JASCO Corporation, Tokyo, Japan) equipped with a single-reflection diamond attenuated total reflection (ATR) accessory. The spectra were measured from 4000 to 600 cm^–1^ with a resolution of 4 cm^–1^ at 32 scans. The PBAT/PLA pellets, the extruded filaments with and without triglycine, and the printed specimens were evaluated. Specimens were analyzed as they were received in each case.

### Mechanical Properties Studies

Mechanical properties such as tensile modulus (*E*), tensile strength (σ), and elongation at break (ε) of the obtained 1BA-shape standard specimens were investigated using a computerized mechanical testing machine model Instron 4204 equipped with a 1 kN load cell, under standard atmospheric conditions at room temperature and by ISO 527:2019.^37^ A static tensile test was performed uniaxially at a strain rate of 20 mm·min^–1^, until the specimen ruptured. Three tests were carried out for each specimen.

### Filament Processing

Filaments containing PBAT/PLA and triglycine as well as pure PBAT/PLA were produced using a single-screw filament extruder (Extrudex, Mühlacker, Germany) with a nozzle that was 3 mm in diameter. The PBAT/PLA pellets were dried in an air-circulating oven at 60 °C for 6 h before the filaments. The required amount of triglycine was mixed with the pellets, and the resulting mixture was fed into the extruder via a hopper. The screw speed was set at 32 rpm and rolling speed at 16.5 min^–1^. These parameters were adjusted by changing the rotational speed of the winding roller to produce filaments with diameters of 1.74 ± 0.02 mm. The temperature profile of the device in the direction of the hopper to the head was as follows: 100–105–110–115–120 °C. The melt was pressed through a nozzle and cooled down before the filament was made and was then spooled. The total retention time of the material in the device was approximately 53 s. In total, three spools of about 100 m were processed in each material (PBAT/PLA with and without triglycine). The presence of triglycine in the material was confirmed by mass spectrometry analysis of the solution after solvent extraction (in acetonitrile with formic acid) of the oligopeptide from the polymer matrix (Figure S1).

### Prototype Printing

PBAT/PLA specimens with two printing temperatures (*T* = 190 °C for pure PBAT/PLA specimen—E190 and PBAT/PLA specimen with triglycine—EG190 as well as *T* = 155 °C for pure PBAT/PLA specimen—E155, and PBAT/PLA specimen with triglycine—EG155) were fabricated using a MEX (Creality Ender-3 3D Printer, Shenzhen Creality 3D Technology Co, Ltd. Shenzhen, China). Specimens according to ISO 527:2019 standard^37^ with dimensions *x* = 12.5 mm, *y* = 80 mm, and *z* = 2 mm were fabricated as shown in Figure 1.

**Figure 1:**
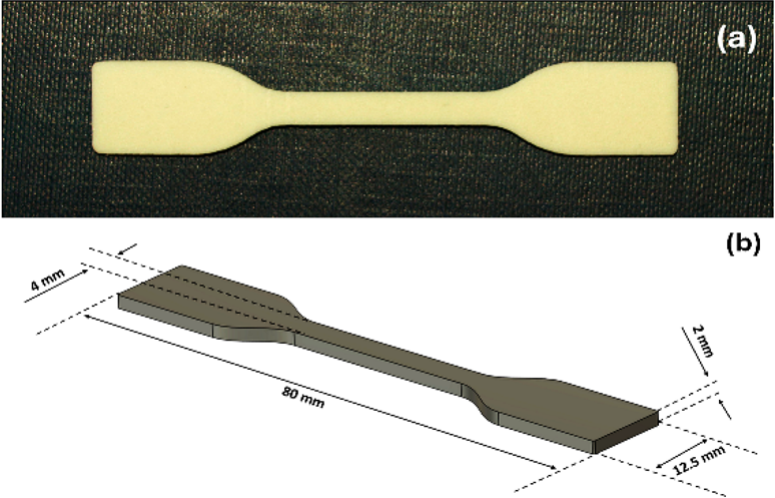
Photo (a) and the CAD model (b) of the PBAT/PLA 1BA-shape standard specimens.

The print parameters of the specimens are listed in Table 1. In this study, print parameters were adapted from a previously reported study, with slight modifications.^30^ The emphasis was to examine the operational temperature range for PBAT/PLA specimens, specifically identifying the minimum temperature at which defect-free prints could be achieved. Printing below 155 °C was unfeasible due to the melting points of the PLA blend component (*T*_m_ = ∼150 °C). While literature shows nozzle temperatures as high as 220 °C, a lower temperature was selected in this case to avoid reaching the thermal decomposition threshold of triglycine, which typically begins around 230 °C, as indicated by TGA analysis and the higher temperatures can lead to polymer degradation, especially the PLA blend component.^38^ The build platform temperature was set to 30 °C and was consistent with prior PBAT/PLA studies, though platform temperatures of up to 60 °C were reported.^30,39^

**Table 1:** Print Parameters for 3D-Printed PBAT/PLA 1BA-Shaped Standard Specimens

3D printing parameters	value	unit
infill pattern density	100	%
layer height (thickness)	0.2	mm
flow rate	100	%
nozzle diameter	0.60	mm
printing speed	110	mm·s^–1^
printing temperature	155 and 190	°C
print bed (building platform) temperature	30	°C

Several specimens with the same parameters were printed at different printing temperatures of 155 and 190 °C using MEX technology. All specimens were fabricated in the same way and in the same print direction but with different temperatures from filaments containing PBAT/PLA and the triglycine. Pure specimens of PBAT/PLA without triglycine were also printed in a similar manner using the same print parameters. All specimens were made from a 1.74 ± 2 mm diameter filament and were printed with the lowest (*T* = 155 °C) and highest (*T* = 190 °C) temperatures that were possible to maintain stable printing conditions at this temperature. To characterize these specimens, several different studies were carried out using several different techniques.

## Results and Discussion

### Structural Analysis of PBAT/PLA Pellets and 1BA-Shaped Standard Specimens

ATR-FTIR spectroscopy is an easy-to-use technique commonly employed to characterize various chemical and physical parameters of polymeric materials.^40,41^ Concerning polymer blends, the most typical property that can be determined based on the infrared spectra is their chemical composition. Since the absorption of infrared radiation by a substance depends on its physical state, it is also possible to identify the crystalline forms and to determine the crystallinity of a specific polymer.^41,42^ It is well-known that PLA is a polymorphic polymer that can exist in four crystalline modifications, namely α, α′ (sometimes denoted by δ), β, and γ.^43,44^ The most important ones are α and α′ forms because they grow under typical crystallization conditions, that is, during crystallization from a molten state, a glassy state, and in a solution. Typically, the less-ordered α′ phase is formed first, and then, it can turn into the more stable α phase after heating above 120 °C. Figure 2 shows the FTIR spectra of the PBAT/PLA pellets and their specimens obtained by 3D printing at *T* = 155 °C (E155 and EG155) and *T* = 190 °C (E190 and EG190). Since the mass of the triglycine in the EG155 and EG190 specimens is only 0.2%, this compound does not give rise to very evident absorption bands in the corresponding spectra.

**Figure 2:**
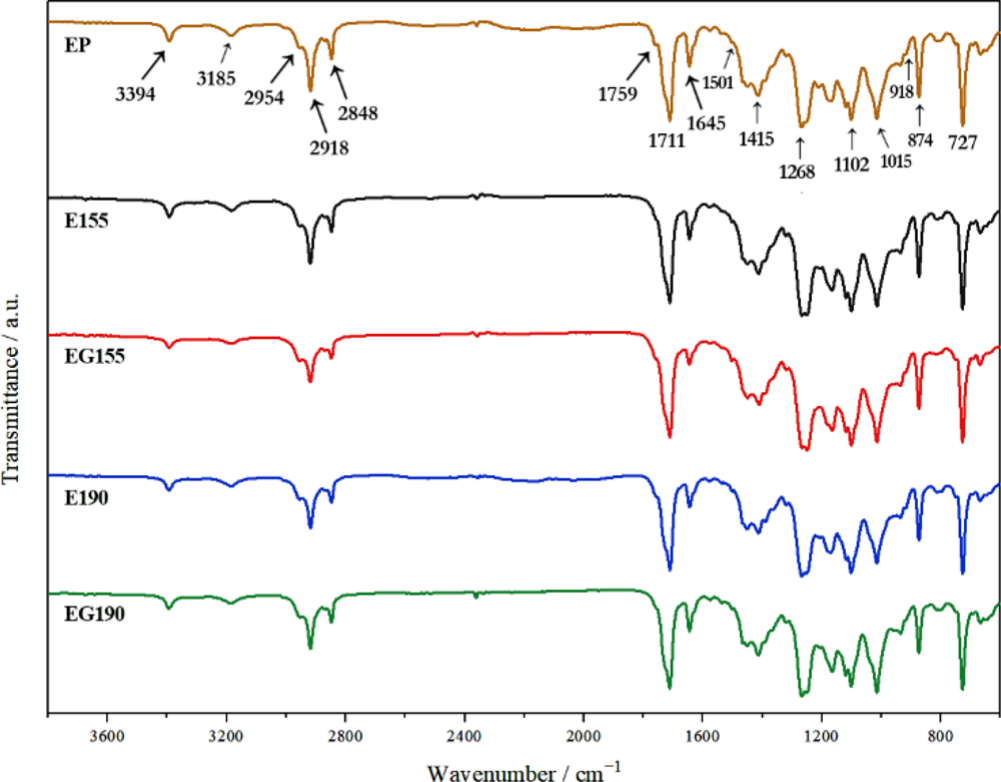
ATR-FTIR spectra of the PBAT/PLA pellets (EP) and AM specimens without (E155 and E190) and with triglycine (EG155 and EG190). Each spectrum was normalized by dividing each intensity value by the average intensity of the absorption bands in the region of 1800–700 cm^–1^; E, Ecovio; G, triglycine; 155 and 190 °C, printing temperatures.

The absorption bands characteristic of PLA and PBAT can be observed in all of the spectra shown in Figure 2. The absorption bands in the 3000–2800 cm^–1^ region can be attributed to the symmetrical and asymmetrical C–H (sp^3^ and sp^2^) stretching of methyl, methylene, and methine groups in PLA and PBAT.^41,43,45^ The peak near 3394 cm^–1^ was reported by Tsou and co-workers as stretching vibrations of the O–H groups in PBAT.^46^ However, this band and the band at 1645 cm^–1^ may as well be due to the vibrations of water molecules.^47^ The water in the specimens may come from talc, a mineral composed of hydrated magnesium silicate that absorbs moisture well. It is an inorganic filler added to the PBAT/PLA pellets to, for example, enhance its crystallinity or improve properties. The stretching mode of the carbonyl groups of PLA and PBAT appears at 1759 and 1711 cm^–1^, respectively. A strong band at 727 cm^–1^ can be ascribed to the out-of-plane bending vibration of the C–H groups of the *p*-substituted benzene ring of PBAT. The benzene ring of PBAT also gives rise to bands at 1501 and 874 cm^–1^. Absorption bands in the 1500–1000 cm^–1^ region are primarily attributed to the bending vibrations of CH_3_, CH_2_, and CH groups and the C–O–C stretching of the ester bonds of PLA and PBAT. These bands, however, highly overlap with each other, so it is difficult to assign them individually.

### Thermal Properties

TGA provides insight into the thermal stability and degradation behavior of materials, enabling a precise understanding of their thermal properties and the optimization of processing conditions. As shown in Figure 3, the degradation of triglycine starts at 230 °C and it is fully degraded at 260 °C, which indicates its thermal stability during filament processing and printing. The TGA curves of the standard PBAT/PLA 1BA-shaped specimens printed at two different temperatures indicate several mass loss steps between 100 and 800 °C.

**Figure 3:**
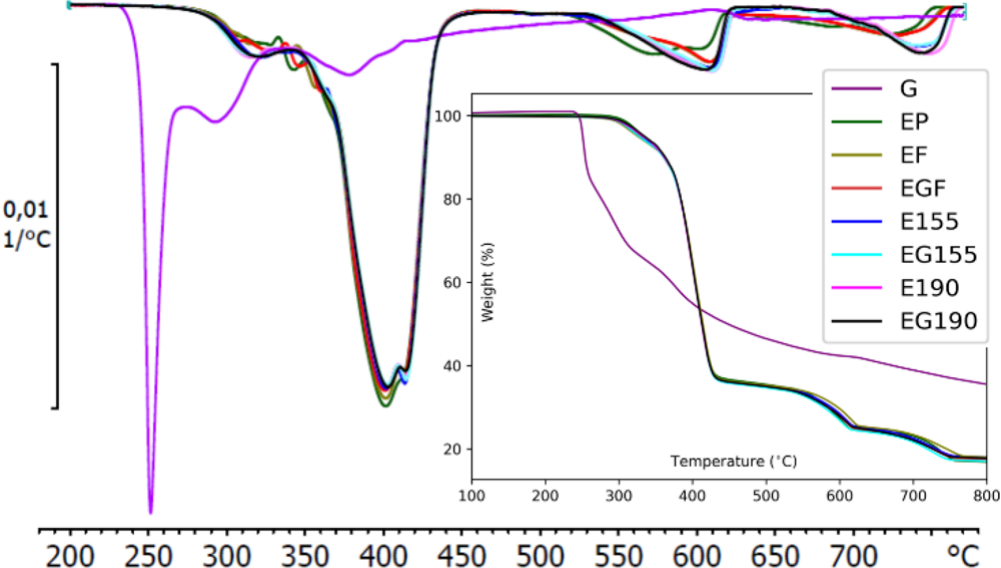
Comparative plots of TGA and DTG curves corresponding to triglycine (G), extruded PBAT/PLA filament without (EF) and with triglycine (EGF), for PBAT/PLA 1BA-shaped standard specimens of E190, EG190, E155, and EG155 obtained by 3D printing; E, Ecovio; G, triglycine; EF, filament; and 155 and 190 °C, printing temperatures.

The first phase of degradation is attributed to PLA, characterized by an initial degradation temperature (*T*_onset_) of around 280 °C, which continues to a final degradation temperature (*T*_endset_) close to 380 °C, with the maximum rate of thermal degradation (*T*_max_) occurring around 320 °C. The second mass loss, which has a *T*_max_ of around 380 °C and *T*_endset_ at 450 °C, is attributed to PBAT components since it exhibits greater thermal stability due to its aromatic structure and longer polymer segments that are much more entangled.^48^ This thermal behavior is consistent with established results in the literature, confirming that PLA and PBAT degrade at different temperatures, each exhibiting unique thermal degradation profiles.^49^ However, the sequential degradation behavior of the structural components of PBAT was observed. As illustrated in Figure 3, the DTG analysis reveals that the decomposition of PBAT takes place in two separate temperatures, where the aromatic part of 1,4-butylene terephthalate units is represented at *T*_max_ ≈ 402 °C for specimens AM at both temperatures and with and without the triglycine, decomposed first.^50^ This is followed by the aliphatic segments with *T*_max_ ≈ 413 for 1,4-butylene adipate units, a result which is consistent with the literature.^51,52^ It is worth mentioning that the PBAT/PLA used in this study was a commercial blend of PBAT that contained 47 mol % of aromatic segments as reported.^22^ Subsequent degradation steps in both TGA and DTG are associated with the presence of commercial additives incorporated by the manufacturer to improve the properties or performance of the material, which also influences the degradation behavior. The thermal degradation behavior of AM PBAT/PLA specimens generally displayed minimal variation. Interestingly, a pronounced difference in thermal behavior emerges when specimens are subjected to temperatures exceeding 500 °C, particularly before and after 3D printing. This deviation suggests the presence of an additive within the filaments, which may undergo chemical or structural changes during the 3D printing process, thereby influencing the degradation properties of the material.

Figure 4 shows the DSC traces with a rate of 20 °C min^–1^ of triglycine, the PBAT/PLA pellets, the extruded PBAT/PLA filament without and with triglycine, and the specimens (with and without triglycine) printed at selected temperatures of 155 and 190 °C.

**Figure 4:**
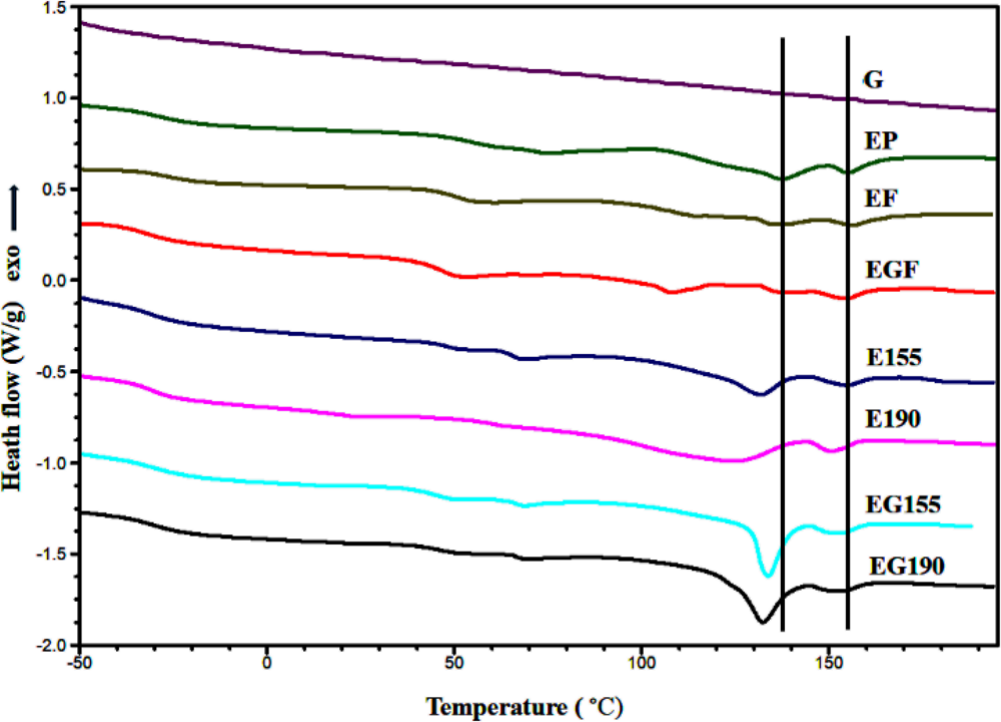
DSC curve overlay of the first heating run (at 20 °C·min^–1^) for PBAT/PLA pellets (EP) and the filaments without (EF) and with triglycine (EGF) as well as the AM PBAT/PLA 1BA-shaped standard specimens of E190, EG190, E155, and EG155; EP, Ecovio; G, triglycine; F, filament; and 155 and 190, printing temperatures.

As shown in Figure 4 (and Figures S2 and S3 in the Supporting Information), the triglycine looks to be stable and shows no sign of degradation up to the maximum temperature range that was used for printing. On the other hand, the PBAT/PLA blends are characterized by two separate glass transition temperatures with regions associated with the *T*_g_ of the PBAT and PLA polymer, respectively. This indicates that the blends are immiscible and present a two-phase structure, due to the significant difference in molecular chain segments and poor compatibility between PBAT and PLA, as has been well documented in the literature.^53^ In addition, previous research shows that the melting range of PBAT and the cold crystallization (*T*_cc_) of PLA are within a similar temperature range.^52^ This means that these two phenomena are occurring at the same temperature, and as a consequence of the overlap of the energetically opposite processes in the same temperature region, it is not practical to calculate the degree of crystallinity of PLA in blends. The overall DSC characteristics of the PBAT/PLA specimens are summarized in Table 2, which shows the *T*_m_ and *T*_cc_ for all specimens in the first and second heating run as well as *T*_g_ in the second heating run.

**Table 2:** Calorimetric Parameters PBAT/PLA 1BA-Shaped Standard Specimen Obtained by 3D Printing at *T* = 155 and 190 °C as well as the Pellets (EP) and the Filament Used^a^

specimen	G	EP	EF	EGF	E155	EG155	E190	EG190
first heating run (20 °C/min)								
*T*_cc_[°C]		103.0	89.3	80.0	92.5	93.9	89.2	97.8
*T*_m_[°C]		137.4/155.0	137.6/156.5	107.9/137.1/154.0	131.7/154.4	133.8/153.6	132.8/151.7	132.2/154.3
Δ*H*_cc_[J g^–1^]		1.54	3.78	2.36	4.69	5.98	2.52	5.65
Δ*H*_m_[J g^–1^]		9.85	5.78	5.28	4.91	5.70	6.53	6.08
second heating run after rapid cooling (20 °C/min)								
*T*_g1_[°C]	169.1	–27.3	–30.2	–28.80	–30.0	–32.3	–30.7	–32.7
*T*_g2_[°C]		61.3	60.9	59.2	59.1	59.6	57.6	59.2
Δ*T*_g_		88.6	91.1	87.8	89.1	91.9	88.3	91.9
*T*_cc_[°C]			54.9/82.4	47.7	52.6		51.8	42.9
*T*_m_[°C]		127.7/153.8	126.9/155.6	121.3/152	118.3/151.6	126.5/151.1	125.5/150.6	128.1/150.8
Δ*H*_cc_[J g^–1^]			3.67	4.39	4.35		3.07	1.36
Δ*H*_m_[J g^–1^]		10.32	9.48	14.71	10.84	10.45/1.11	13.42	5.72/1.19

a E, Ecovio; G, triglycine; F, filament; 155 and 190, printing temperatures. *T*_*m*_, melting temperature; *T*_*g*_, glass transition temperature; *T*_*cc*_, maximum of the exothermic peak of the cold crystallization temperature; Δ*T* = (*T*_gPLA2_ *– T*_gPBAT1_) represents the degree of compatibility, Δ*H*_cc_ is cold crystallization enthalpy, Δ*H*_*m*_ is melting enthalpy.

The *T*_*g*_ of the PBAT/PLA pellets is the highest among all the blend components, measured at −27.3 °C for PBAT and 61.3 °C for PLA (Figure 4 and Figures S4, S5, and S6 in the Supporting Information). For the extruded filament (Figure 4 and Figures S7 and S8 in Supporting Information), a slight reduction in *T*_*g*_ was noted compared to the pellets, with a further decrease observed in the *T*_*g*_ of the filament with the triglycine (Figure 4 and Figures S9, S10, and S11 in the Supporting Information). MEX of the 1BA-shaped standard specimens, incorporating the triglycine, led to an even greater reduction in *T*_*g*_, particularly for the PBAT component, reaching −32.7 and −32.3 °C for the EG155 and EG190 specimens, respectively (Figure 4 and Figures S12, S13, S14, S15, S16, and S17 in the Supporting Information). Consequently, this results in a lowered *T*_*g*_ of the triglycine-containing specimens suggesting its effect, as the *T*_*g*_ reflects the point at which polymer chains gain mobility, and the addition of triglycine increases the flexibility, softness, and mobility of the polymer. Table 2 also highlights Δ*T*_*g*_, representing the difference in glass *T*_*g*_ between PLA and PBAT. This parameter serves as an indicator of the compatibility between the two polymers and the effect of the triglycine, where smaller Δ*T*_*g*_ values indicate improved miscibility. The Δ*T*_*g*_ values calculated showed that the E155 and the E190 have lower Δ*T*_*g*_ values (Figure 4 and Figures S18, S19, S20, S21, and S22), while both EG155 and EG190 have similar values, which are slightly higher due to the addition of the triglycine. The Δ*T*_*g*_ is within a narrow range of 87.8 to 91.9 °C, with a maximum variation of only Δ*T*_*g*_ = 3.5 °C. This minimal difference in *T*_*g*_ between specimens is in good agreement with the results of previously published studies.^52^ The theoretical *T*_*g*_ value of the blend can be calculated using the Fox equation.^54^ The Fox equation is also commonly applied to predict the glass transition temperature in polymer blends. This can also highlight the effect of the additive as well as the compatibility of the blends, as the presence of a low-molar mass additive increases the free volume of the system and subsequently lowers *T*_g_. Fully miscible blends would exhibit a single *T*_*g*_, as their morphological properties directly influence their thermal behavior.^55^

From the DSC results, two melting points were observed for all specimens as seen in Table 2. The first is related to the *T*_*m*_ of PBAT crystallites, while the second corresponds to the *T*_*m*_ of the PLA component crystallites. The *T*_*m*_ of PLA is around 151–156 °C, and the PBAT has a *T*_*m*_ = 118–132 °C (in the first heating run). Overall, the addition of triglycine did not cause significant changes in the melting temperature of the specimens; however, temperature shifts can be seen from the DSC thermogram, and the enthalpy is higher for those with oligopeptide. In addition, as shown in Figure 4 and Table 2, the *T*_*cc*_ of the 1BA-formed PBAT/PLA standard specimen containing triglycine was significantly lower than for specimens without triglycine. This reduction is attributed to the lubricating effect of triglycine, which likely facilitates molecular mobility during the crystallization process.^56^

### PBAT/PLA 1BA-Shaped Standard Specimens’ Mechanical Characteristics

To assess the performance of AM parts, mechanical property studies play a crucial role. Figure 5 shows the analysis of the mechanical properties of the E155, EG155, E190, and EG190 specimens printed by MEX.

**Figure 5:**
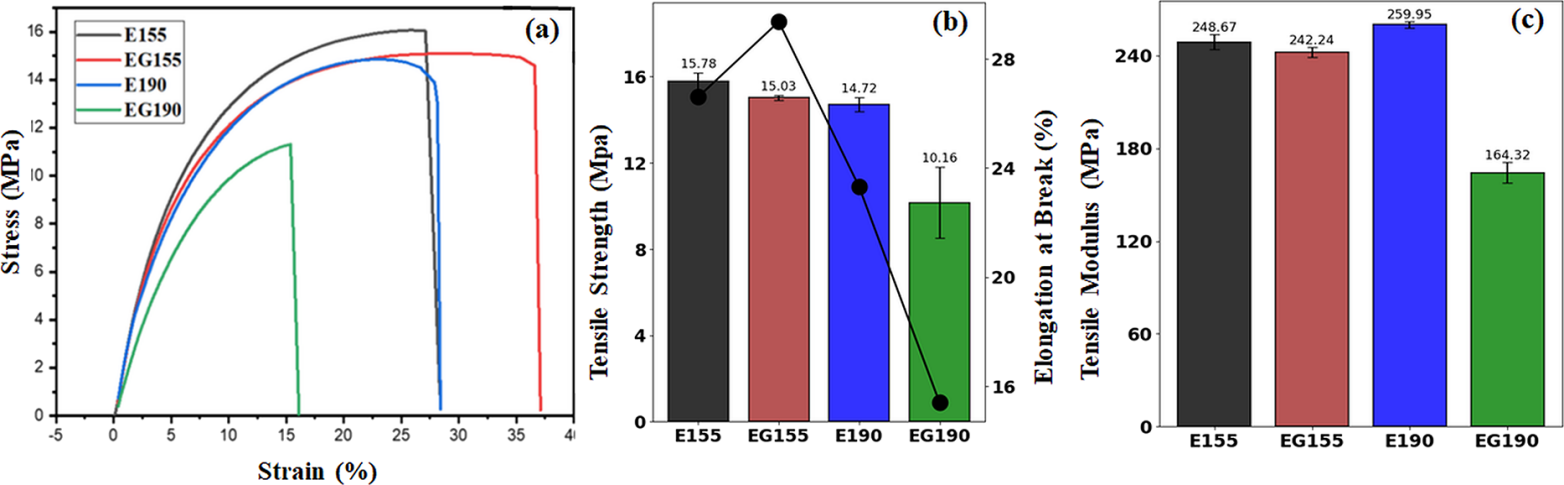
Representative stress–strain curves (a), ultimate tensile strength and elongation at break (b), and tensile modulus (c) were determined for AM PBAT/PLA 1BA-shaped standard specimens of E190, EG190, E155, and EG155; E, Ecovio; G, triglycine; and 155 and 190 °C, printing temperatures.

The analysis focuses on key metrics such as the ultimate tensile strength (*F* ^tu^), *E*, and ε. These are important parameters for understanding the behavior of the material under load and ensuring the structural integrity of printed components. The results obtained reflect the influence of both printing temperature and the addition of the triglycine, which acts as a plasticizer in this case. The mechanical properties of PBAT and PLA differ significantly due to their inherent structural characteristics. The stress–strain curve indicates that the shift from elastic to plastic deformation is not sharply defined, and ductile fracture is due to the highly flexible nature of the PBAT component. Specimen E155, printed at a lower temperature (*T* = 155 °C) without triglycine, shows the highest *F* ^tu^ = 15.78 ± 0.41 MPa and *E* = 248.67 ± 4.89 MPa, showing that the lower print temperatures preserve the material strength.

The slight reduction in *F* ^tu^ = 15.03 ± 0.11 MPa for EG155, which includes triglycine but is printed at the same temperature (*T* = 155 °C), is balanced by a significant increase in elongation at break (ε = 29.35%) compared to the ε = 26.6% of E155, highlighting the plasticizing effect of the triglycine. On the other hand, E190, printed at a higher temperature (*T* = 190 °C) without triglycine, exhibits slightly lower *F* ^tu^ = 14.72 ± 0.33 MPa compared to both E155 and EG155 but a higher *E* = 259.95 ± 2.26 MPa compared to E155, which are printed at a lower temperature. This reflects the typical effect of increased print temperature, where higher thermal exposure can reduce tensile strength due to partial degradation but improve stiffness by reducing voids and improving merging between layers during printing. PBAT is known for its flexibility, exhibiting a high elongation at break of about 508%, while PLA, on the other hand, is a stiff and brittle polymer with a much lower elongation at break of around 6.5%.^57^

The presence of the triglycine in EG190, printed at the same higher temperature (*T* = 190 °C) as E190, results in the most severe reduction in mechanical properties, with a *F* ^tu^ of 10.16 ± 1.65 MPa and *E* of 164.32 ± 6.86 MPa, along with a significantly lower ε = 15.4%. This sharp deterioration can be attributed to the combined effects of plasticization and thermal degradation at higher temperatures, which exacerbate the already poor interfacial compatibility between PLA and PBAT.^53^

The mechanical properties of 3D-printed objects are influenced by the print parameters and material composition. Parts produced by MEX AM often exhibit low tensile strength due to defects such as voids, interlayer gaps, and poor interlayer adhesion, which degrade mechanical properties. Yu et al.^38^ investigated PLA/PBAT blends with PBAT contents ranging from 0 to 40 wt %, produced using a twin-screw extruder, to assess their mechanical performance. Their results showed a significant decrease in the tensile strength of PLA with increasing PBAT content, with the tensile modulus dropping from 24 MPa for the 80/20 PLA/PBAT sample to 17 MPa for the 40 wt % PBAT sample. Similarly, the observed tensile modulus decreased from 26 MPa in the 80/20 sample to around 14 MPa at 40 wt % PBAT. Moreover, a recent study showed that the elastic modulus was the property most affected by variations in the 3D printing parameters. Increasing temperature and print speed increased stiffness, with a peak of 17.11 MPa reached at 210 °C.^39^ However, when the print speed was held constant, the temperature variation had no statistically significant effects. This is consistent with our results, where temperature variations from 155 to 190 °C for specimens without triglycine produced no noticeable differences in the mechanical properties of the specimens.

### Morphological Observation

Figure 6 (and Figures S23, S24, S25, and S26 in the Supporting Information) shows representative SEM micrographs of cryogenically fractured surfaces of the PBAT/PLA specimens.

**Figure 6:**
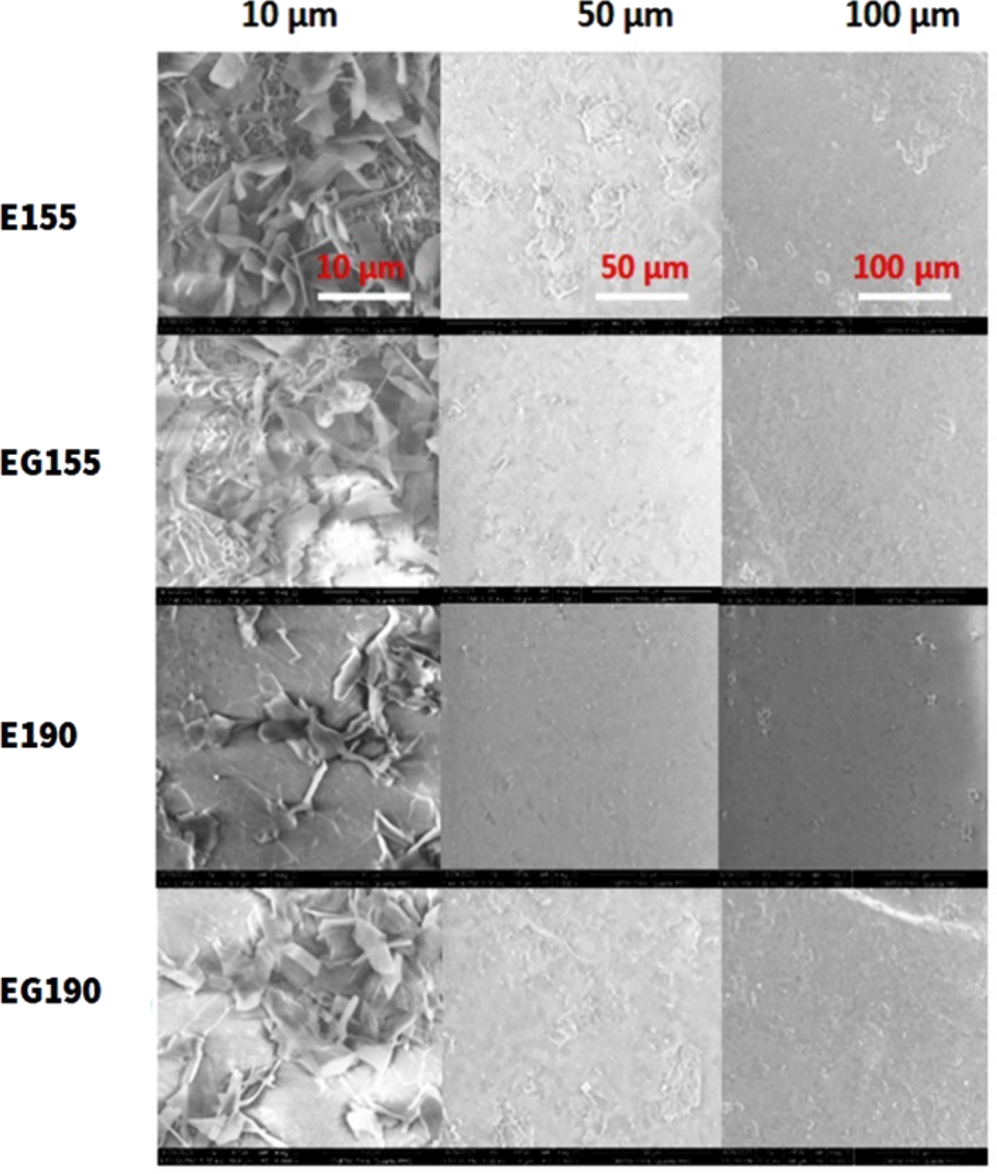
Selected representative SEM micrographs of PBAT/PLA 1BA-shaped standard specimens of E190, EG190, E155, and EG155 obtained by 3D printing; E, Ecovio; G, triglycine; and 155 and 190 °C, printing temperatures.

The surface analysis of all specimens revealed a consistent pattern characterized by a homogeneous and smooth structure, formed of fused layers without visible voids. The morphology of the EG190 specimen showed slightly rougher fractured surfaces and a small, discrete PLA spherical domain or droplet structure within the PBAT matrix. The E155 specimen also exhibited some spherical domain features such as EG190. This is characteristic of PBAT/PLA blends with higher PBAT content and is in line with previously reported observations.^54^

In the case of immiscible polymer blends such as PBAT with PLA, the domain size of the dispersed phase is influenced by several factors, including the component ratio, processing conditions, and interfacial tension.^54,58^

## Conclusions

In this study, an experimental investigation of the printability and temperature effects of PBAT/PLA containing a small addition of triglycine (0.2 wt %) was conducted. The work highlights the versatility of AM as a platform for the fabrication of biodegradable polymer systems with customized functionalities. Of particular interest here is the development of blends based on aliphatic–aromatic biodegradable copolyesters and PLA, which show significant potential in a wide range of applications. Despite their favorable properties, the use of such blends in the context of AM is still relatively unexplored. This research further investigates the incorporation of triglycine as a functional additive in PBAT/PLA matrices with the aim of modulating the physicochemical properties and extending the applicability of these materials in extrusion-based AM techniques.

The DSC result indicates that the addition of triglycine did not significantly affect the *T*_*m*_ of the specimens; however, it reduced the disparity in glass transition temperature between PLA and PBAT. SEM images do not indicate significant morphological changes, which suggests that the quality of the printed specimens is adequate even for those printed at 155 °C. This is a novel achievement in itself, as the literature usually reports PBAT/PLA specimens printed at nozzle temperatures of over 180 °C and the lowest nozzle temperature reported for PBAT/PLA specimens in the literature was 170 °C.^27^ Mechanical studies revealed that PBAT/PLA filaments exhibit significant potential for creating stable 3D structures, even at a relatively low printing temperature of 155 °C. This lower temperature of 155 °C was deliberately chosen in this study to assess the stability of the specimens for applications with thermally sensitive active substances that cannot withstand high temperatures. Moreover, a correlation was identified between the print temperature and triglycine content, with AM specimens containing triglycine displaying the lowest ultimate tensile strength and tensile modulus. The triglycine addition improved elongation at break, as EG155 undergoes significant deformation before breaking, compared to the pure PBAT/PLA specimens printed at the same temperature. In contrast to this result, though, a substantial decrease in tensile strength was observed for the specimens with triglycine printed at *T* = 190 °C compared to those printed at *T* = 155 and 190 °C without triglycine. The deterioration of the mechanical properties can be attributed to a weakening of the interfacial interaction between PLA and PBAT at elevated temperatures. From this, it can be concluded that the findings indicate that triglycine promotes plasticization in PBAT/PLA specimens at both processing temperatures but accelerates thermal degradation at higher temperatures. Ongoing studies aim to validate these effects, with a comprehensive degradation analysis, and will be presented in a forthcoming publication.

## Supplementary Material



## Notes

DSC traces and additional SEM images for all tested materials (PDF)

## †

SPIN-Lab Centre for Microscopic Research on Matter, University of Silesia in Katowice, 75 Pułku Piechoty 1A, 41–500 Chorzów, Poland

## #

Department of Chemistry, Himachal Pradesh University, Shimla, 171005, India
