# Supplementary material for: Preparation and Characterization of Triglycine-Containing 3D-Printed PBAT/PLA Specimens
Source: ACS Omega. 2025 May 26;10(22):23817–26. doi: 10.1021/acsomega.5c03205 (PMC12163685; doi:10.1021/acsomega.5c03205)
Supplement: Supplementary file 1 [file ao5c03205_si_001.pdf]

# Supporting Information

# Preparation and characterization of triglycine containing 3D-printed PBAT/PLA specimens

Khadar Duale*‡, Alexander Grundmann§, Simon T. Kaysser§, Sönke Detjen§, Paweł Chaber‡, Jakub Włodarczyk‡, Henryk Janeczek‡, Marta Musioł‡, Iza RadeckaΦ, Marek Kowalczuk‡, Anna Hercog‡†, Sunita Ranote‡†† and Joanna Rydz‡

‡Centre of Polymer and Carbon Materials, Polish Academy of Sciences, M. Curie-Skłodowskiej 34, 41-800 Zabrze, Poland

§CompriseTec GmbH, Rödingsmarkt 20, 20459 Hamburg, Germany

ΦSchool of Life Science, Faculty of Science and Engineering, University of Wolverhampton, Wulfruna St., Wolverhampton WV1 1LY, UK

* Corresponding author: : (K.D.) kduale@cmpw-pan.pl

Supporting Information contains 12 pages and 25 figures

Mass spectrometric analysis

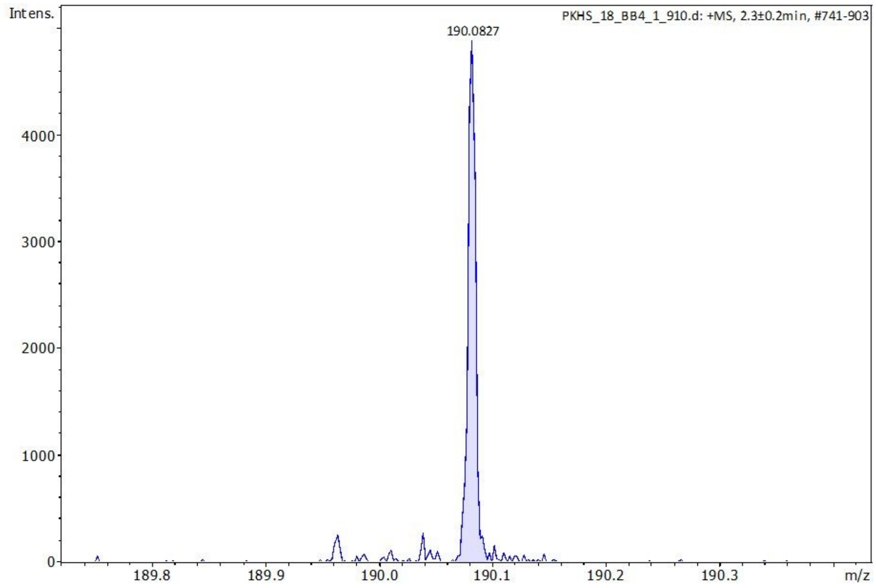


Figure S1. Mass spectrometric analysis of the solution after solvent extraction from the polymer matrix after 3D printing. The $[M + H]^+$ peak at *m/z* = 190.082 ± 3 ppm corresponds to monoisotopic pseudo-molecular ion.

Differential Scanning calorimetry (DSC) curves for all the samples

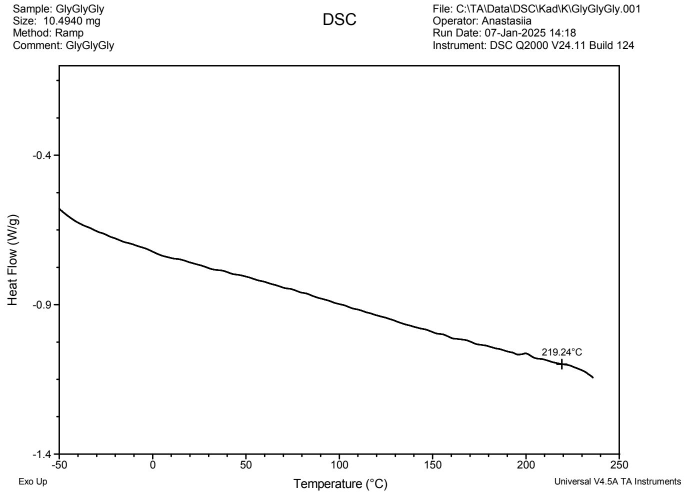


Figure S2. Original DSC curve of the first heating run (at 20 °C/min) for G – triglycine.

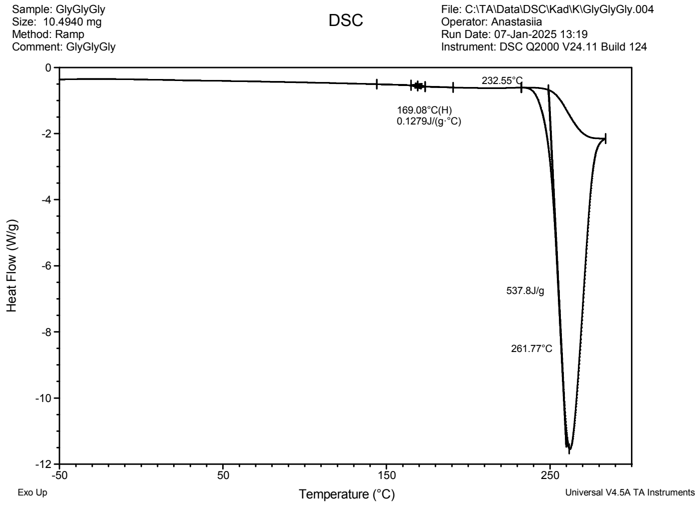


Figure S3 Original DSC curve of the second heating run (at 20 °C/min) for G – triglycine. $T_g$= 169.1°C, $T_d$=261.8°C decomposition.

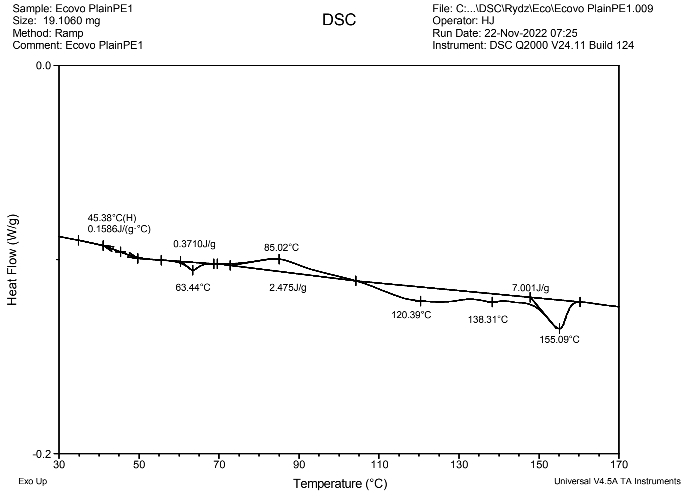

Figure S4 Original DSC curve of the first heating run (at 2.5 °C/min) for PBAT/PLA pellets (EP).

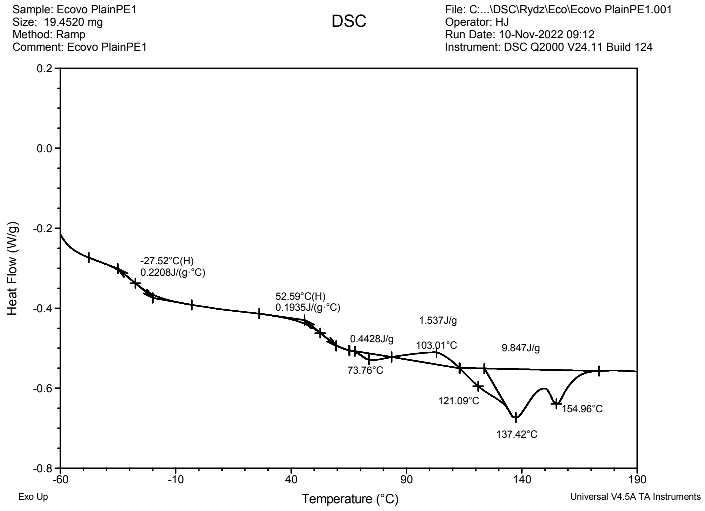

A

Figure S5 Original DSC curve of the first heating run (at 20 °C/min) for PBAT/PLA pellets (EP).

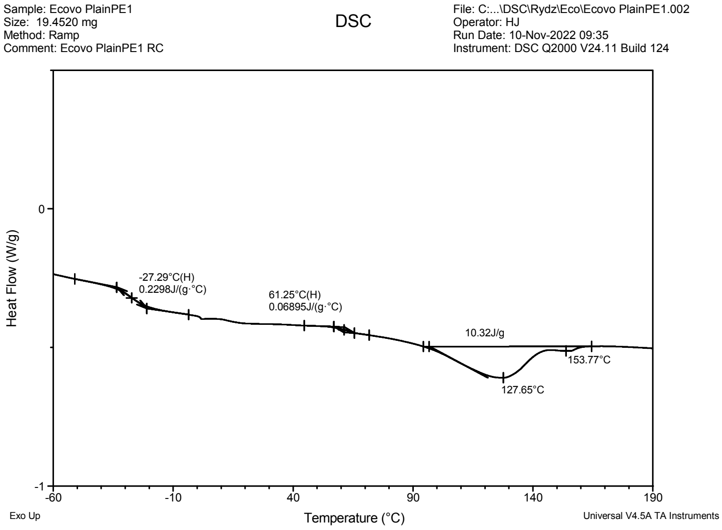

B

Figure S6 Original DSC curve of the first heating run (at 20 °C/min) for PBAT/PLA pellets (EP).

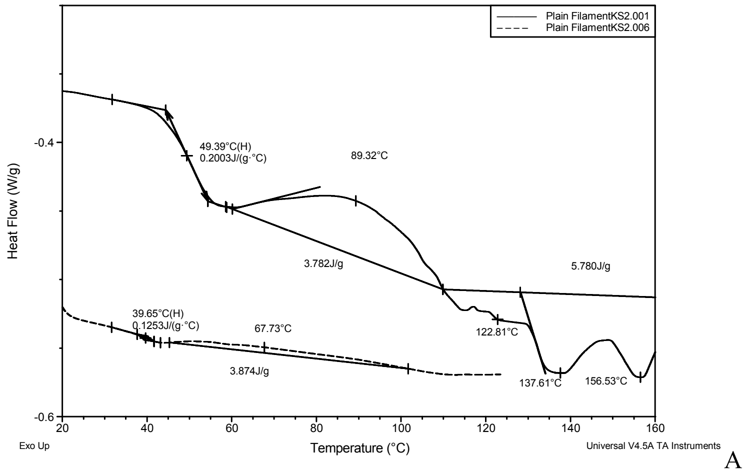


A

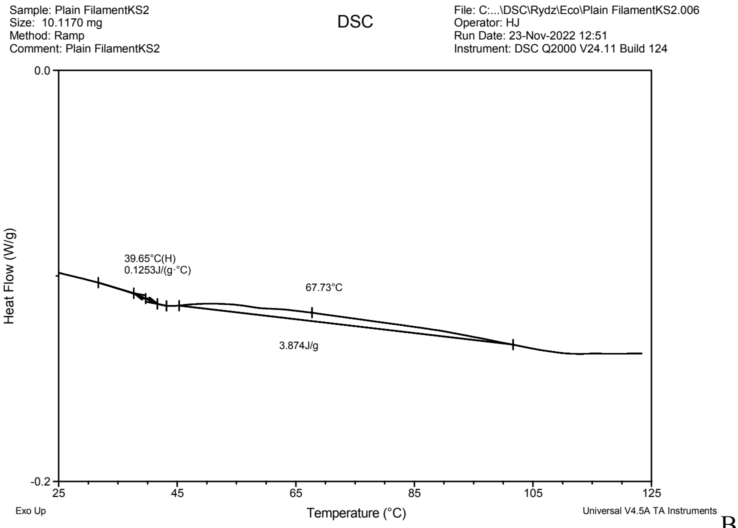


B

Figure S7 Original DSC curve of the first heating run (at 2.5 °C/min) for the filaments without (EF).

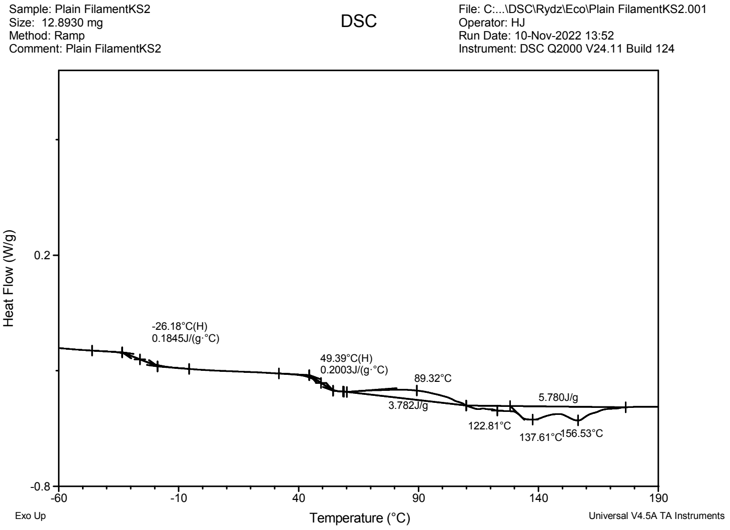


Figure S8 Original DSC curve of the first heating run (at 20 °C/min) for the filaments without (EF).

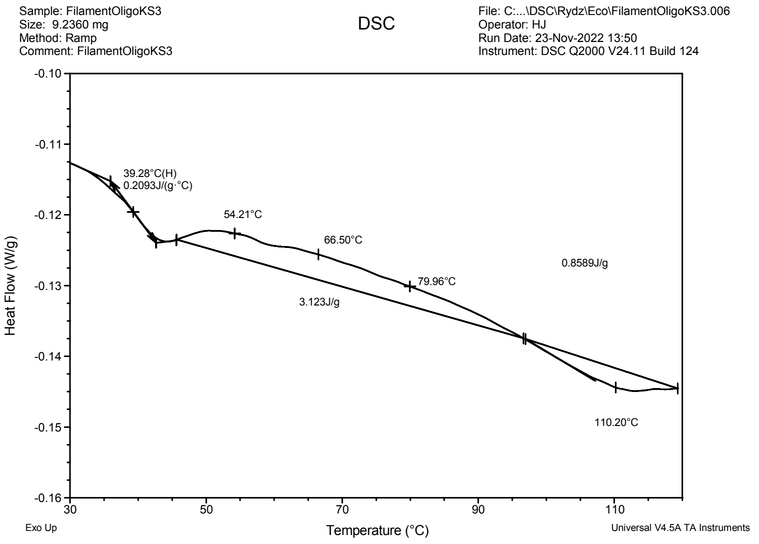


Figure S9 Original DSC curve of the first heating run (at 2.5 °C/min) for the filament with triglycine (EGF).

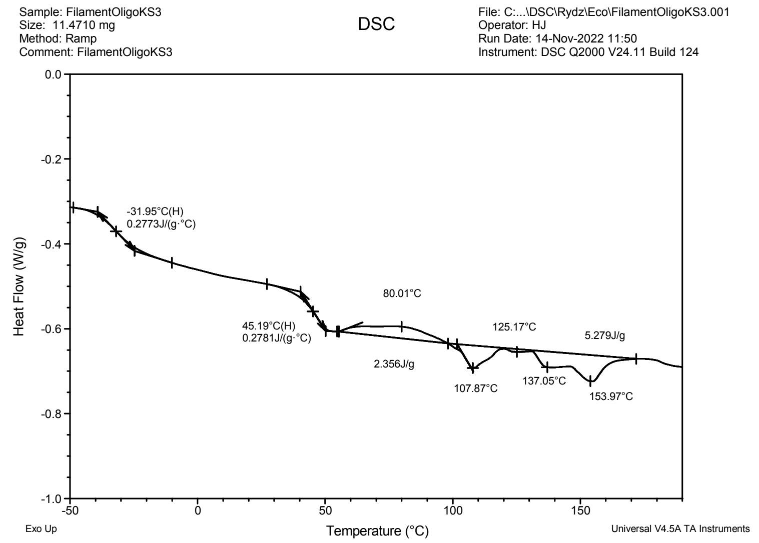


Figure S10 Original DSC curve of the first heating run (at 20 °C/min) for the filament with triglycine (EGF).

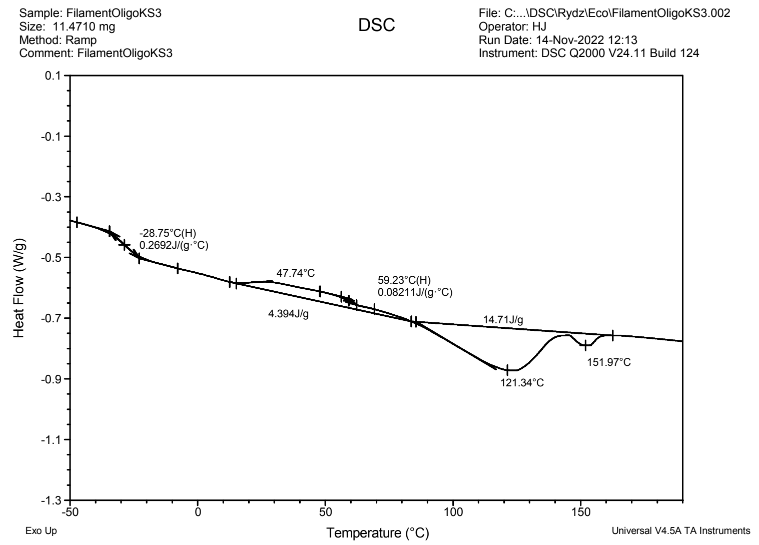

Figure S11 Original DSC curve of the second heating run (at 20 °C/min) for the filaments with triglycine (EGF).

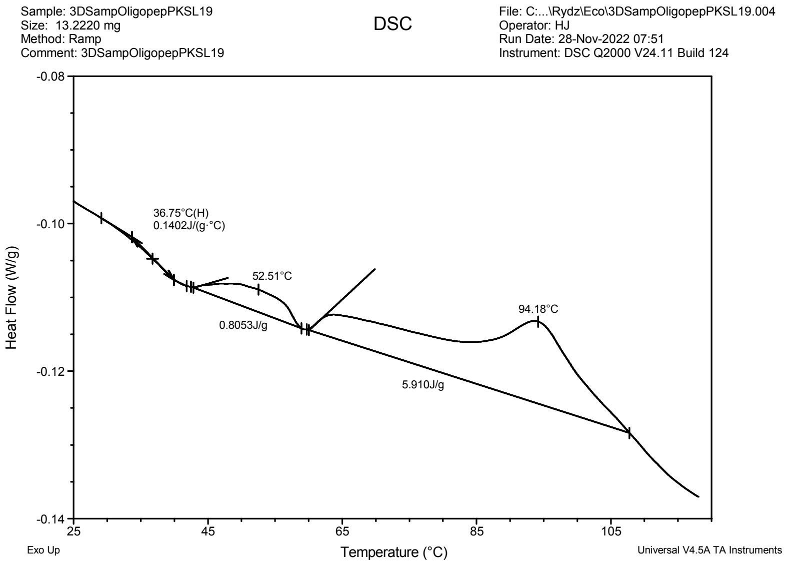


Figure S12 Original DSC curve of the first heating run (at 2.5 °C/min) for AM PBAT/PLA 1BA-shape standard specimen of EG155.

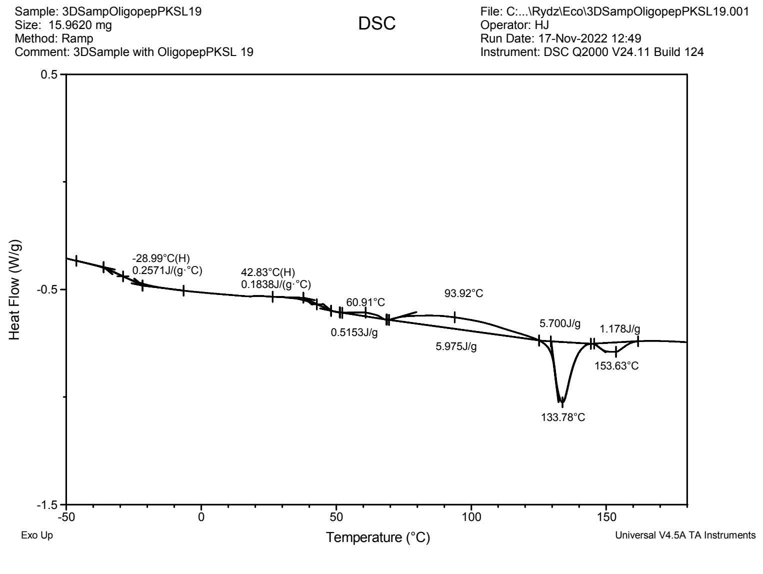


Figure S13 Original DSC curve of the first heating run (at 20 °C/min) for AM PBAT/PLA 1BA-shape standard specimen of EG155.

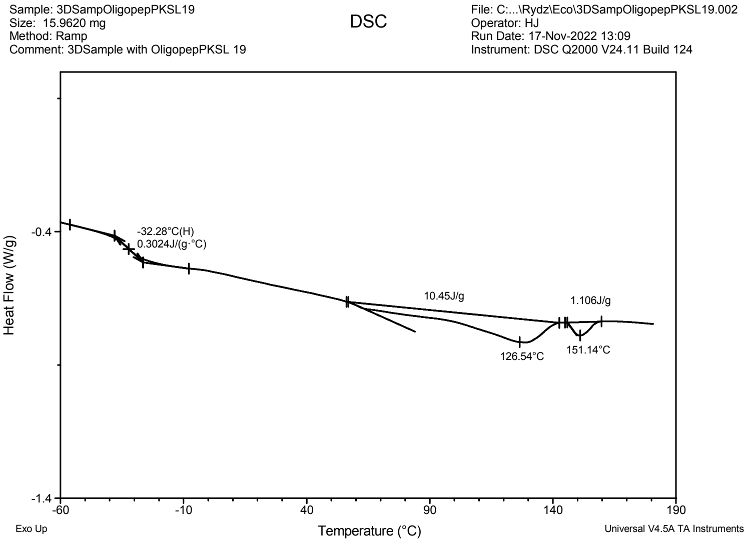


Figure S14 Original DSC curve of the second heating run (at 20 °C/min) for AM PBAT/PLA 1BA-shape standard specimen of EG155.

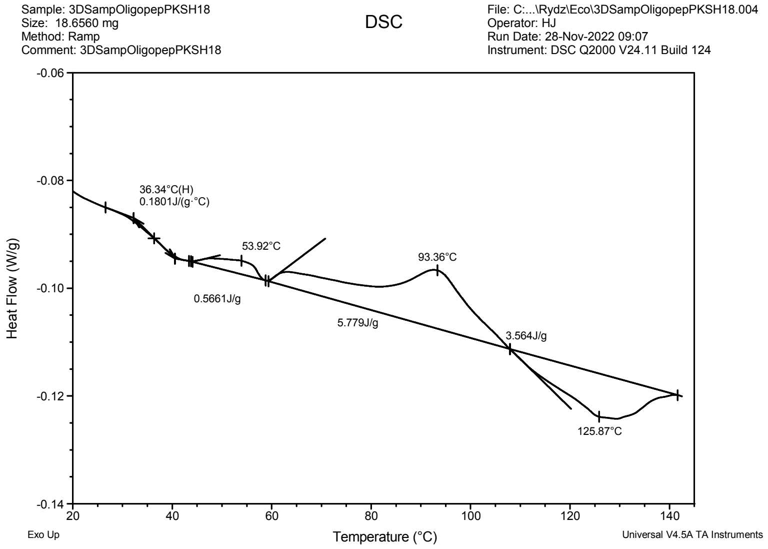


Figure S15 Original DSC curve of the first heating run (at 2.5 °C/min) for AM PBAT/PLA 1BA-shape standard specimen of EG190.

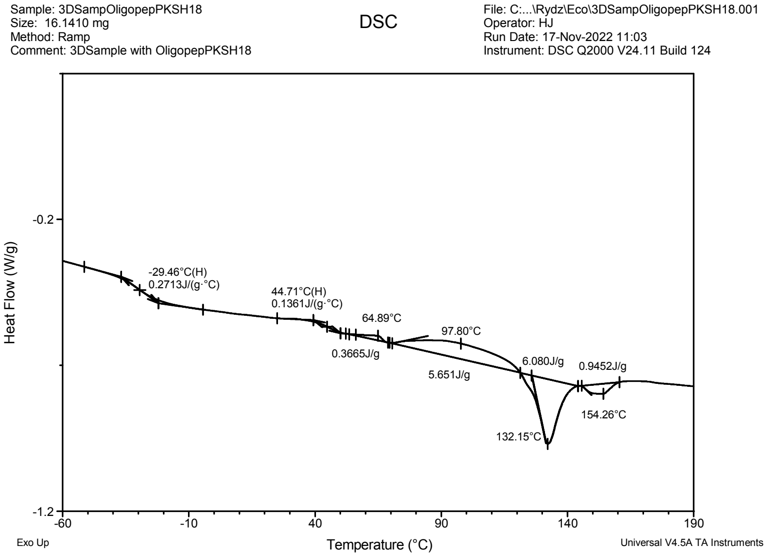


Figure S16 Original DSC curve of the first heating run (at 20 °C/min) for AM PBAT/PLA 1BA-shape standard specimen of EG190.

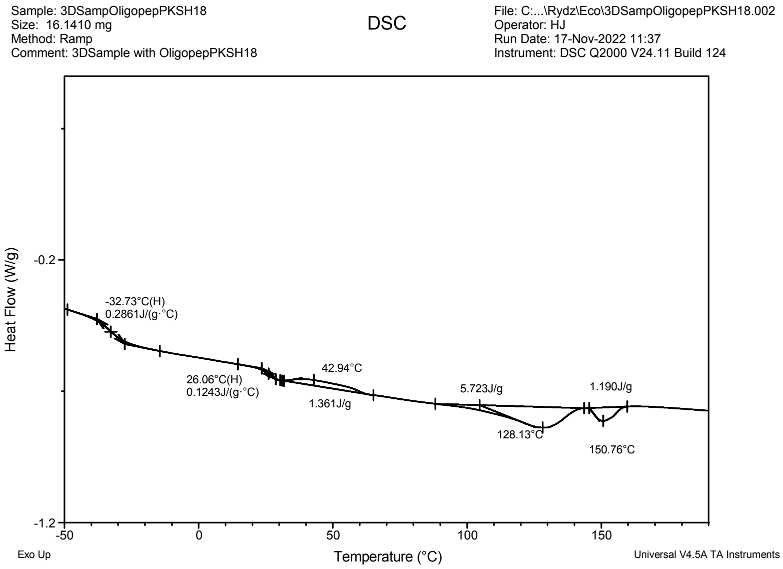


Figure S17 Original DSC curve of the second heating run (at 20 °C/min) for AM PBAT/PLA 1BA-shape standard specimen of EG190.

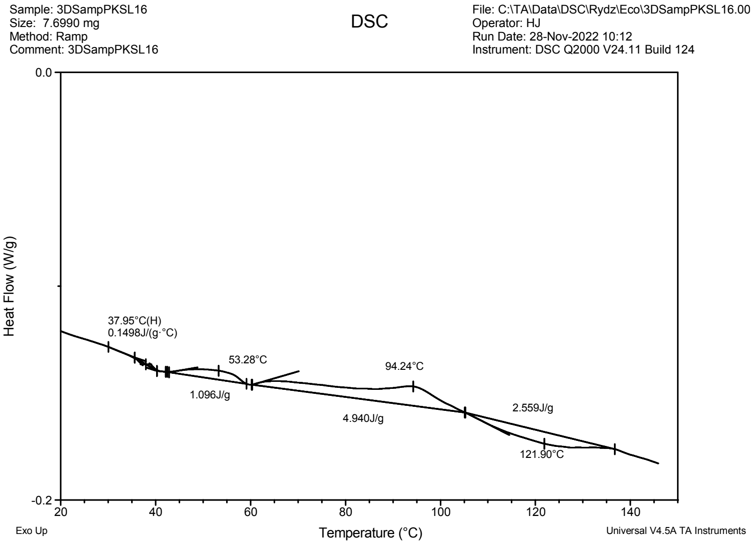


Figure S18 Original DSC curve of the first heating run (at 2.5 °C/min) for AM PBAT/PLA 1BA-shape standard specimen of E155.

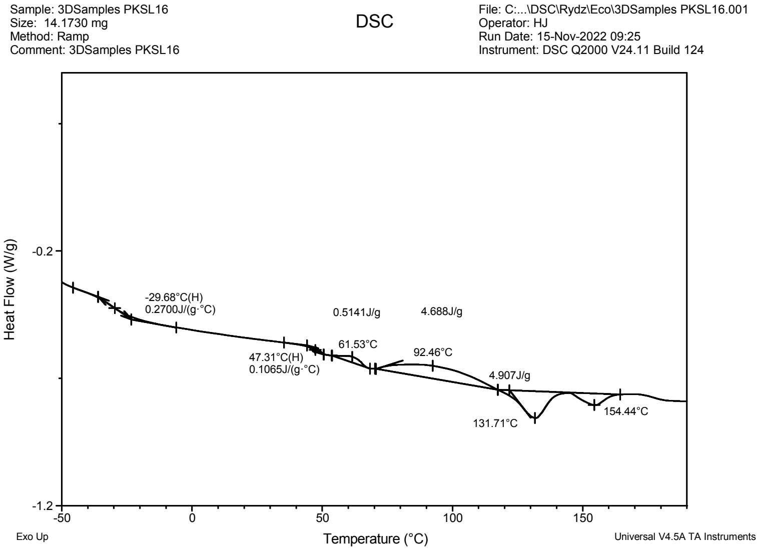


Figure S19 Original DSC curve of the first heating run (at 20 °C/min) for AM PBAT/PLA 1BA-shape standard specimen of E155.

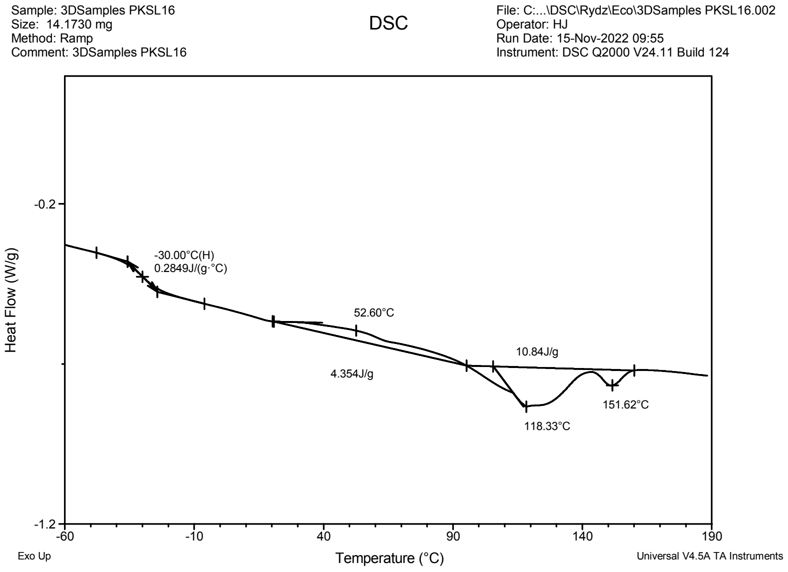


Figure S20 Original DSC curve of the second heating run (at 20 °C/min) AM PBAT/PLA 1BA-shape standard specimen of E155.

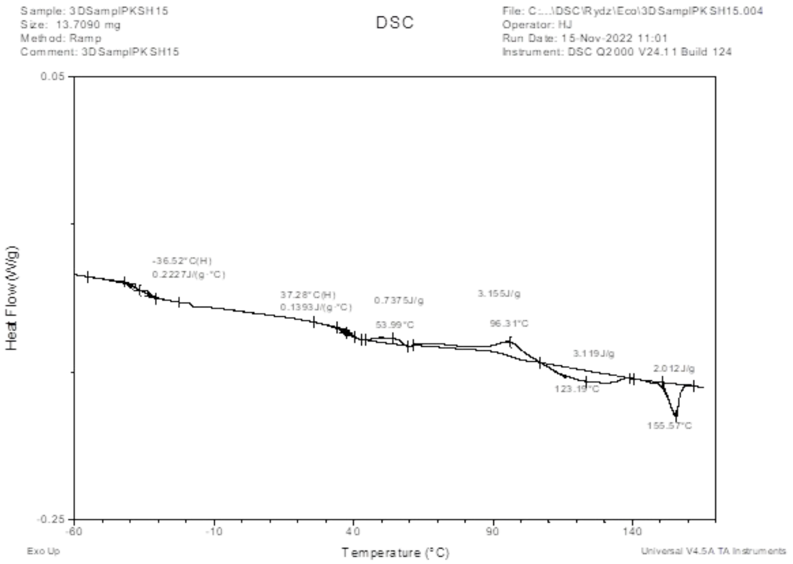


Figure S21 Original DSC curve of the first heating run (at 3 °C/min) for AM PBAT/PLA 1BA-shape standard specimen of E190.

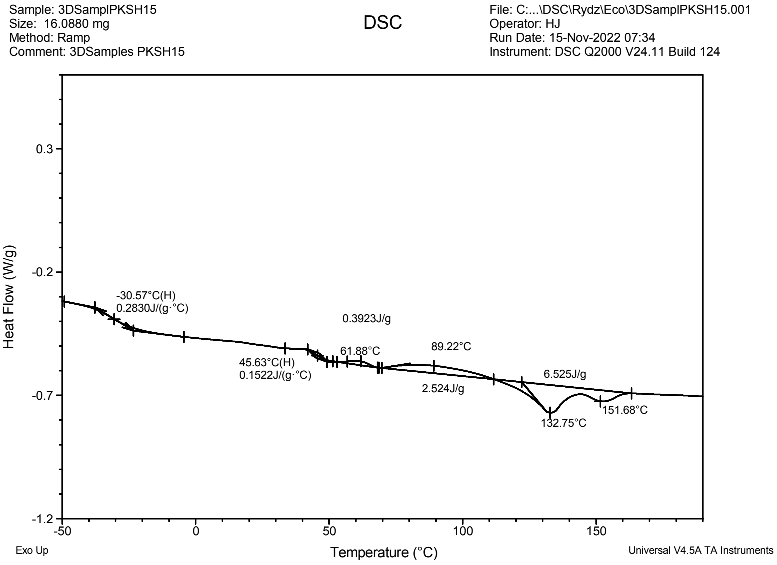


Figure S22 Original DSC curve of the first heating run (at 20 °C/min) for AM PBAT/PLA 1BA-shape standard specimen of E190.

SEM micrographs for all AM samples

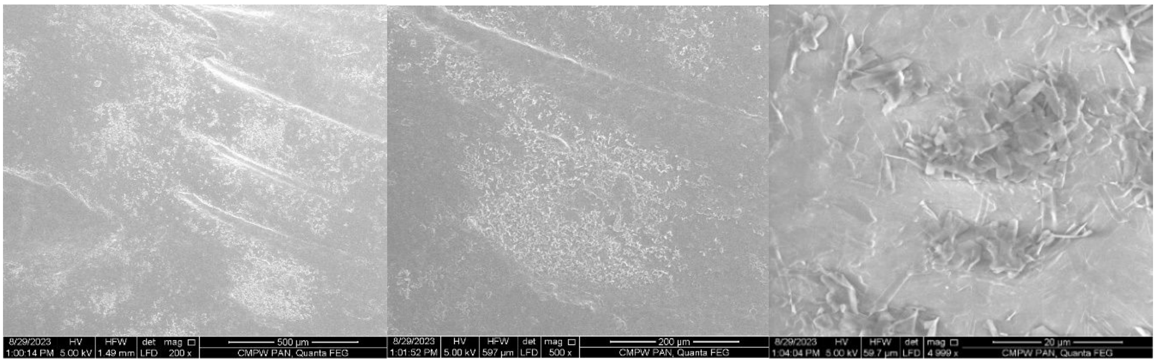

Figure S23 Representative SEM micrographs of PBAT/PLA 1BA-shape standard specimens of E155 obtained by 3D printing at 155°C – printing temperatures.

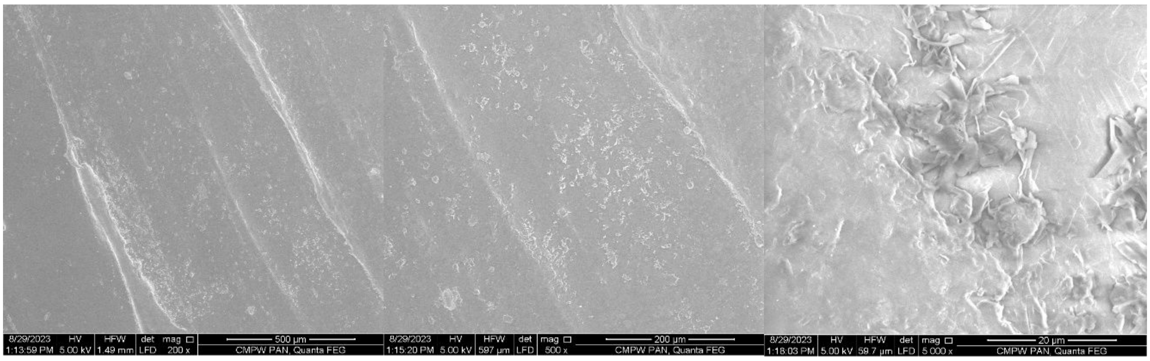

Figure S24. Representative Figure 6. Selected representative SEM micrographs of PBAT/PLA 1BA-shape standard specimens of EG155 obtained by 3D printing at 155 °C – printing temperatures.

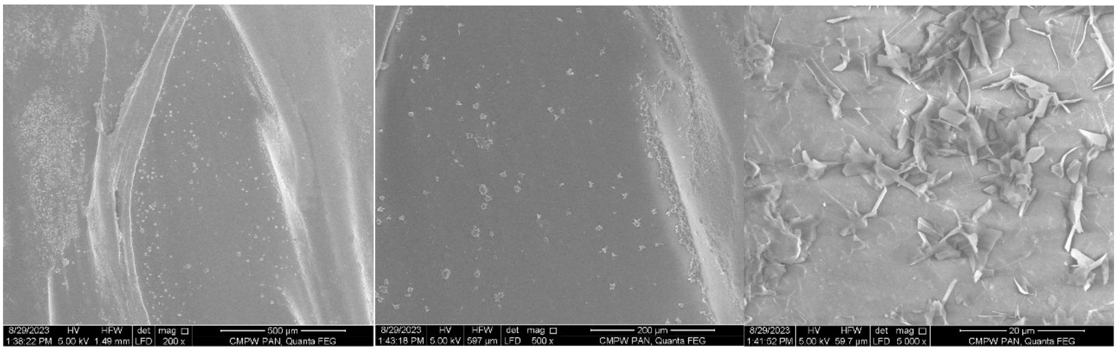

Figure S25. Representative SEM micrographs of PBAT/PLA 1BA-shape standard specimens of E190 obtained by 3D printing at 190 °C – printing temperatures.

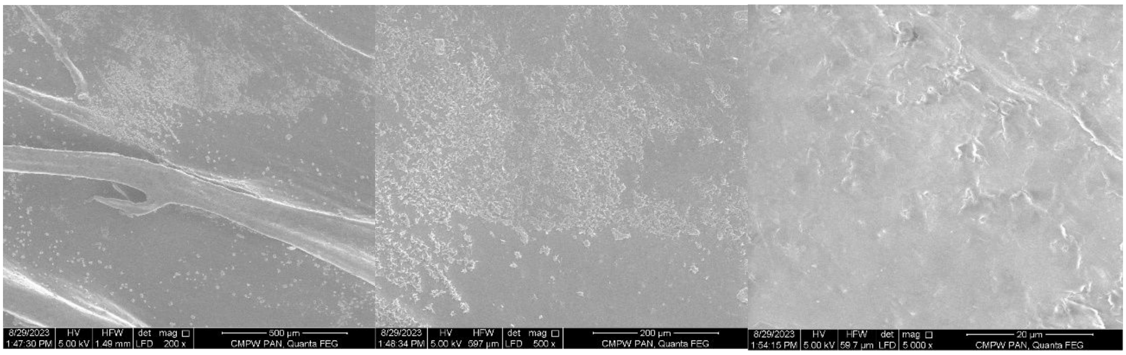

Figure S26. Representative SEM micrographs of PBAT/PLA 1BA-shape standard specimens of EG190 obtained by 3D printing at 190 °C – printing temperatures.
